# Heating Apparatus

**Published:** 1873-04

**Authors:** 


### HEATING APPARATUS.

The first impression made upon the reader of this heading, would likely be that the article is inopportune and out of season; not so, however, for the winter having passed, the perplexities and annoyances attending the usual heating appliances are fresh in our memory, and the summer is the proper season in which to make repairs or to remove defective apparatus and to supply its place with better. It is very bad management to wait until the approach of cold weather reminds us that our furnaces or stoves should be placed in order for winter service.—This being the usual habit of people, stove dealers are kept very busy in making the necessary changes, and it is with great difficulty that their services can be obtained at the required time. Summer is the proper season to make such preparations, when the workmen can discharge their duties to a much better advantage, and the household is not rendered uncomfortable through the open doors and the delays necessaary upon such occasions.

The time for making changes and repairs having been disposed of, what sort of an apparatus shall we adopt? Before deciding upon this point, let us consider the several means usually adopted for heating purposes. First of all, we have the ordinary stove, the most generally adopted plan for heating the house, as it is the most unhealthful and uncomfortable. By the stove, we heat only the air that is confined in the room, which, having passed over the surface of the hot iron, becomes heated, rises to the ceiling, cools, falls to the lower portion of the room, passes through our lungs, where it becomes poisoned with carbonic acid gas, is again heated, and again breathed, until the occupants are compelled to open a window or door to prevent absolute suffocation. The ingress of pure cold air affords relief, until it becomes heated and poisoned through the same process. Any one, it appears to us, can discover at a glance, that this is a miserably defective means of heat ing an apartment in which to live.

The hot-air furnace is an improvement upon this means, as the cold air is supplied from out doors, through a cold-air box, passes over the radiating surface of the hot cylinder of the furnace, and thence by means of tin conducting pipes to the living rooms above. This is better—much better than the stove plan, for the reason only, that the same air is not continually re-heated and re-breathed, and the volume of heat so produced being so great, that suitable ventilation is provided at the *bottom* of the room for the escape of the vitiated air. But the great and insurmountable difficulty attending a hot-air furnace is the escape of noxious gases into the hot-air chamber of the furnace, and from thence into the house. This occurs when the flue is partially closed to check the draft of the furnace; when two gases, the result of the combustion of anthracite coal—carbonic acid and carbonic oxide mixed with sulphurous acid gas—instead of passing up the chimney, escape into the cellar, and from thence into the house, through the floors and hot-air conductors. These gases escape still more plentifully after the furnace has become old and warped by the constant burning it is subjected to. But no matter how tight the joints may be, hydrogen, carbonic oxide, carbonic acid, and other highly deleterious gases pass *through* the red hot iron of the cylinder, and mix with the heated air that is supplied to our lungs. Then, again, the intense heat to which the pure air from out doors is subjected, by passing over the furnace cylinder, robs it of a portion of its oxygen, the life sustaining property of the air we breathe, by literally burning it up; and were it not for the bountiful supply that reaches us through the cold-air box, our condition would be as bad as when in an apartment heated by the stove.—Again, the heat so produced, is too intense; if you will place your thermometer in the hot air chamber of your furnace, you will find, unless it is an unusally long one, that it is incapable of registering the degree to which the heat attains. It will reach from 1200° to 2000°. Such a heat drives every particle of moisture from the air, so that it is re-supplied as far as can be, from the walls of the room, and from the bodies of the inmates. It is for this reason that the skin feels hot and parched, for every particle of moisture almost, is literally sucked out of it, and we involuntarily rush from the room, or open wide the windows or doors for relief. Placing pans of water in the furnace for evaporation, partially overcomes this difficulty, by giving rise to another, that of causing the vapor to condense upon your walls, curtains and pictures, over which it runs in little rivulets, to stain whatever it comes in contact with, or to afford a settling place for the dust which arises at every shake of the furnace grate.

Much time and money has been expended by scientific men, within a few years, to overcome the objections to the hot-air furnace above cited. The one that comes nearest to being perfect, was invented by Dr. Nichols, the chemist, of Boston. It is made entirely of wrought iron, with joints so thoroughly rivited as to prevent the escape of gases or dust into the hot-air chamber. Yet, the objection still exists that these gases will pass through the hot iron itself, or, when the damper in the flue is partially closed, into the cellar.

There remains, therefore, but one means yet devised, for warming our houses with pleasant and wholesome heat; namely, by steam. We have been to much expense in testing several different kinds of hot-air furnaces in our dwelling, and having become thoroughly disgusted with them, from the imperfections above enumerated, we directed our attention to the system of steam-heating, with a hope of overcoming these difficulties and supplying our home with a heat-producing apparatus that shall be at once healthful, economical and durable.

The hot-water apparatus requires but little notice: it is noisy and leaky, and insufficient in extremely cold weather. The steam-heating plan, when accomplished through radiators, stationed in the living-room, is objectionable, for the reason that the air heated, although of a pleasant warmth, is only supplied by the room; therefore becomes noxious from being rebreathed, unless good ventilation is provided, in which case, the heat is not intense enough to keep the apartments comfortable in the coldest of our winter weather. The process would answer better in such apartments as are not continually occupied; but for the living rooms,—parlor, sitting and dining rooms, nothing but fresh air, from out doors, properly and comfortably heated, minus the gases and dust that naturally arise from the combustion of coal, will answer; and such a machine we have found. It is named the "Union Steam and Water Heating Apparatus," and is erected by E. H. Cook & Co., of this city. It is not within the province of this article to enter into a description of this remarkable apparatus; a very elegant little treatise descriptive of it, and containing much valuable information upon the subject of heat and ventilation, is published by the above mentioned firm, and which can be had free of charge, by making application for it. What we desire to do is to point out a few of the excellencies of this particular system of heating. Firstly and prominently, it furnishes pure air, from out doors, heated to a temperature of 212°, by being passed over cylinders of hot water, instead of over red-hot iron, as in the hot-air furnace. By this means the air is not thrown in contact with the gases of the fire-box, is not burned and deprived of its oxygen and moisture, but is as pure as when first introduced to the heating surface, and the heated air furnished is so abundant that proper means of ventilation can be adopted,—that of having suitable openings in the floor through which the carbonic acid gas, exhaled from the lungs, and which always falls to the floor, can be allowed to escape, without preceptably lowering the temperature of the room through the admission of any cold air that might reach the apartment through the same channel.

It may not be generally known, yet such is the fact, that moist air is warmer than dry.—This is accounted for in the fact that insensible perspiration is constantly taking place on the body when under the influence of dry heat,—such as is furnished by a hot-air furnace, which lowers the temperature of the skin to that degree as to require a much higher temperature in the room to satisfy the demands of comfort.—In other words, a room heated by air passing over cylinders of hot water, at a temperature of 212°, is much warmer, and infinitely more healthful and comfortable, than the burnt air coming from red-hot iron at 1500°.

Another advantage that the steam possesses over the hot-air apparatus, is its automatic regulator, which can be so adjusted as to keep a perfectly even temperature of higher or lower degree, as may be required by the temperature of the weather. This regulator acts automatically, regulating the draft to the fire-box, by the pressure of the steam, and is accomplished by closing the lower draft, *through* the coal, causing it to pass *over* its surface, and out at the chimney, carrying with it all the products of combustion, instead of forcing it into the cellar by closing the flue, as is the plan in most hot-air furnaces. This is accomplished by the regulator itself, without the aid of the servant, by an ingenious device, fully described in the little work before referred to.

We could point out many other excellencies of the steam heating apparatus, but these already described we deem of the most importance, and which alone recommend it to every intelligent reader. The cost of erecting such a heater is about double that of an ordinary hot-air furnace; but after once in your building, costs nothing for repairs, much less for fuel, and furnishes a heat at once healthful, delightful and comfortable. It is as far superior to the hot-air furnace, as is the glorious air and sunshine of out-doors, to that of an illy ventilated and crowded apartment.

We have had the pleasure of examining two of these steam heaters in this city, in the large and elegant dwellings of John Arnot, Esq., and Mr. S. T. Reynolds, during the coldest of our winter weather, and were charmed by the delightful manner in which these apartments were warmed. Mr. Arnot, who has tried several hot-air furnaces, has failed utterly in making his large mansion comfortable, but now speaks even more enthusiastically of the steam heater than ourself.

We offer this our opinion and experience in heating apparatus, confidently believing that those of our readers who are dissatisfied with their furnaces, or who contemplate building new homes, will thank us for the information given.

